# Comprehensive Evaluation of Amino Acids and Polyphenols in 69 Varieties of Green Cabbage (*Brassica oleracea* L. var. *capitata* L.) Based on Multivariate Statistical Analysis

**DOI:** 10.3390/molecules26175355

**Published:** 2021-09-03

**Authors:** Ning Jin, Li Jin, Shilei Luo, Zhongqi Tang, Zeci Liu, Shouhui Wei, Fanhong Liu, Xiaoqiang Zhao, Jihua Yu, Yuan Zhong

**Affiliations:** 1 College of Horticulture, Gansu Agricultural University, Lanzhou 730070, China; Jinn0513@163.com (N.J.); jinli0124@163.com (L.J.); Luosl1021@163.com (S.L.); tangzq@gsau.edu.cn (Z.T.); liuzc@gsau.edu.cn (Z.L.); wlj920229@163.com (S.W.); Liufh3962@163.com (F.L.); 2 Gansu Provincial Key Laboratory of Aridland Crop Science, Gansu Agricultural University, Lanzhou 730070, China; zhaoxq3324@163.com

**Keywords:** cabbage varieties, free amino acids, polyphenols, HCA, PCA

## Abstract

The biological activities of the primary metabolites and secondary metabolites of 69 green cabbage varieties were tested. The LC-MS detection method was used to determine the content of 19 free amino acids (lysine, tryptophan, phenylalanine, methionine, threonine, isoleucine, leucine, valine, arginine, asparagine, glycine, proline, tyrosine, glutamine, alanine, aspartic acid, serine, and glutamate). The content of 10 polyphenols (chlorogenic acid, gallic acid, 4-coumaric acid, ferulic acid, gentisic acid, cymarin, erucic acid, benzoic acid, rutin, and kaempferol) was determined by the HPLC detection method. Considering the complexity of the data obtained, variance analysis, diversity analysis, correlation analysis, hierarchical cluster analysis (HCA), and principal component analysis (PCA) were used to process and correlate amino acid or polyphenol data, respectively. The results showed that there were significant differences between the different amino acids and polyphenols of the 69 cabbage varieties. The most abundant amino acids and polyphenols were Glu and rutin, respectively. Both amino acids and polyphenols had a high genetic diversity, and multiple groups of significant or extremely significant correlations. The 69 cabbage varieties were divided into two groups, according to 19 amino acid indexes, by PCA. Among them, seven varieties with high amino acid content all fell into the fourth quadrant. The HCA of amino acids also supports this view. Based on 10 polyphenols, the 69 cabbage varieties were divided into two groups by HCA. Based on 29 indexes of amino acids and polyphenols, 69 cabbage varieties were evaluated and ranked by PCA. Therefore, in this study, cabbage varieties were classified in accordance with the level of amino acids and polyphenols, which provided a theoretical basis for the genetic improvement of nutritional quality in cabbage.

## 1. Introduction

Cabbage (*Brassica oleracea* L. var. *capitata* L.), a member of the Brassica genus of the cruciferous family that includes broccoli, mustard, cauliflower, Brussels sprouts, kale, kohlrabi, and bok choy, is one of the most cultivated cruciferous vegetables worldwide [1]. The large-scale cultivation and promotion of cabbages is mainly on account of the short vegetative cycle, wide adaptability, disease resistance and high productivity of cabbages. In 2016, the Food and Agriculture Organization Statistics Database (FAOSTAT, http://www.fao.org. accessed on 20 June 2021) reported that among the top three of the world’s largest cabbage producing countries are ranked China, India and Pakistan. Various cultivars of cabbage present a great diversity in appearance quality (leaf size, shape and color, head texture, etc.) and internal quality (folic acid, vitamins, flavor substances, etc.) [2,3,4]. Moreover, cabbage is highly favored by people because of its rich and diverse varieties and high nutritional value. It is often used as a raw material for making various cuisines, such as raw vegetable salads, sauerkraut, kimchi and cooking food [5].

Studies have shown that a diet with a high proportion of vegetables is essential to reduce the risk of gastrointestinal diseases and cancer [6]. Cabbage is an important imperative vegetable crop and central food source because it contains many bioactive compounds, such as glucosinolates (GSLs), anthocyanins, amino acids, and polyphenols [7,8]. Plant tissues produce primary and secondary metabolites with different biological functions. Amino acids, as the primary metabolites and basic units of protein, have many prominent functions, which are required for plant growth, development, and reproduction [9]. They provide the basis for other biosynthetic pathways and play a key role in signal transduction, plant stress response, and protein biosynthesis [10]. Nine essential amino acids for human body, including lysine (Lys), tryptophan (Trp), phenylalanine (Phe), methionine (Met), threonine (Thr), isoleucine (Ile), leucine (Leu), and valine (Val), are obtained from food. Nowadays, the scientific community has recognized the benefits of consuming antioxidant rich food supplements, because their presence can trigger the expression of protective enzymes such as catalase or superoxide dismutase, or they can interact with other related proteins such as albumin [11,12]. Amino acids are also precursors of various metabolites and neurotransmitters. As antioxidants, they can also effectively scavenge free radicals [13]. Another important field where amino acids are highly valued as food supplements is in sports. Athletes’ endurance and recovery ability can be improved by eating specific amino acids. Previous studies have also reported the role of some amino acids as biological antioxidants, such as glutamic acid (Glu) and aspartic acid (Asp), which can help reduce oxidative stress [14]. Amino acids are abundant in cabbages, and their contents are important for nutrition.

Phenolic acids and flavonoids, as secondary metabolites widely present in plants, are collectively called polyphenols, which play an important role in the survival and defense of plants [15]. Phenolic acids present in the seeds and peels of various plant-based foods, especially vegetable leaves, such as caffeic acid, chlorogenic acid, erucic acid, ferulic acid, and p-coumaric acid, are due to their inhibition of lipid oxidation and removal. Active oxygen has a strong antioxidant activity [16]. Flavonoids can also act as scavengers of reactive oxygen species and electrophiles in vitro, and as chelating agents of metal ions, so they may help reduce the risk of cardiovascular disease in vivo [17]. Studies have also shown that flavonoids, which are widely present in nature, have a high degree of species diversity, a variety of pharmacological and significant biological activities, such as anti-allergic, anti-inflammatory, anti-oxidant, anti-mutagenic, anti-cancer, and enzyme-regulating activities [18]. Cabbage shows a good source of health-promoting compounds that have a preventive effect on cancer, atherosclerosis, nephritis, and diabetes because it is rich in phytochemicals such as phenolic acids, flavonoids, and GSLs and their hydrolysates [19]. At present, amino acids and polyphenols are the most interesting potential antioxidants and have been widely used in medicine, food, and health products.

In addition to the above-mentioned nutritional and functional effects, amino acids and polyphenols are also considered to be important biomarkers for plant metabolomics and metabolomics research, and their metabolic changes can reflect the response of plants to biotic and abiotic stresses [20,21,22]. Eight amino acids, such as proline and phenylalanine, were up-regulated due to exposure to pesticides, indicating a higher level of oxidative stress [23]. The research on white cabbage after insect damage shows that the primary and secondary compounds, such as phenolic acids and flavonoids, may play an important role in insect resistance [24]. Many studies have mentioned that plants need to endure various abiotic stresses, and polyphenols accumulate under these stresses to help plants adapt to unfavorable environments [25,26]. Hence, the content of amino acids and polyphenols in plant tissues is a good indicator for predicting the tolerance of plants to abiotic and biotic stresses. Under the action of an array of external factors, the tolerance of plants to abiotic and biotic stresses varies greatly with plant species or varieties. Furthermore, changes in amino acids, flavonoids, polyphenols, and vitamins have been found to be related to tomato breeding [27]. In view of previous studies, it is particularly important to clarify the differences in amino acids and polyphenols of different cabbage varieties. The purpose of this experiment is (i) to evaluate the genetic variability of 19 amino acids and 10 polyphenols in 69 cabbage varieties by diversity analysis, correlation analysis, hierarchical cluster analysis, and principal component analysis, and to identify promising varieties with higher amino acids or polyphenols; and (ii) to comprehensively evaluate and rank 69 cabbage varieties based on amino acids and polyphenols by PCA. The research results aim to provide a theoretical basis for consumers’ selection of cabbage varieties and the selection of new cabbage varieties, thereby promoting the genetic improvement of cabbage’s nutritional quality.

## 2. Results and Discussions

### 2.1. Difference Analysis of Amino Acid and Polyphenol Contents in 69 Varieties of Cabbage

#### 2.1.1. Variance Analysis

At present, a variety of methods have been reported to quantitatively determine amino acids, including GC-MS [28], CE-MS [29,30], LC-MS [31,32], and many other methods. Compared with other known methods, the advantage of LC-MS is that amino acids can be analyzed without derivatization. Therefore, this study used the LC-MS technique to determine the content of 19 amino acids in 69 green cabbage varieties. Free amino acids are important parameters that reflect the flavor and nutritional value of fruits and vegetables [33,34]. Some amino acids, such as Asp and Glu, may contribute to a sour taste; Ala, Gly, and Ser are more conducive to a sweet taste [35]; Leu, Phe, Trp, and Tyr are related to a bitter taste [36]; and they are also closely related to human taste perception [37]. Table S1 has shown that there are significant differences in the distribution of free amino acids among various cabbage varieties. In terms of total free amino acids, the top five cabbage varieties with the most abundant content are V34, V30, V33, V35 and V31, and the top five cabbage varieties with the least abundant content are V42, V45, V17, V53 and V64. These results have suggested that the top five varieties rich in total free amino acids may have a better flavor and nutritional value.

Polyphenols not only have anti-allergy, anti-inflammatory, anti-oxidant, anti-mutation, and anti-cancer effects, but have also been tested for the effects on weight status in both animal and human studies. Evidence from in-vitro and randomized controlled trials shows that some polyphenol compounds can also promote the reduction of adipocyte occurrence, differentiation, and proliferation, in addition to preventing inflammation and promoting lipolysis [38]. As a vegetable salad, cabbage is often used as a weight loss food. In our study, there were significant differences in the composition of polyphenols in various varieties of cabbage (Table S2). The five varieties with the most abundant total polyphenol content among 69 cabbage varieties are V23, V6, V41, V28, and V26, which suggests that they may have potential anti-obesogenic effects. The reason for this hypothesis is the potential interaction between polyphenols and gut microbiota. Increasing evidence shows that the presence of phenolic compounds can promote the beneficial effects of intestinal probiotics and inhibit invasive species [39,40]. The five varieties with the lowest content of total polyphenols (V10, V8, V14, V7, and V5) had little effect on anti-obesogenic.

#### 2.1.2. Diversity Analysis

The results showed that the highest content of the 19 amino acids in the 69 varieties of cabbage was Glu, which could reach 12.259 mg/g (Table 1). In the research on *Brassica* spp., Glu has also been proven to be the most abundant amino acid [41], which is consistent with the results of our study. An amino acid analysis of 239 apricot varieties showed that the most abundant amino acid is Gly [36], which is inconsistent with the results of our experiment, but which may be related to the different cultivated species. Polyphenols, especially flavonoids, have not only been shown to have anti-cancer effects [18], but also improve learning and memory processes, and their potential to examine age-related cognitive decline has been studied in both animals and humans [42,43,44,45]. Among the 69 cabbage varieties, the rutin (P9) was found to be the most abundant of the 10 polyphenols, which indicates that these cabbage varieties have a better nutritional value (Table 1). The diversity of amino acids and polyphenols in cabbage adequately reflects the diversity of its genetic background, because the 69 cabbage varieties in this study are consistent in site conditions, cultivation, and management conditions. CV is usually used as the main indicator to characterize the genetic differences between germplasm, representing the diversity of related traits. Generally speaking, the CV of traits with rich genetic backgrounds is also large, which has a high reference significance for germplasm identification and evaluation [46]. CV was used to describe the genetic variation of the mineral element content of 36 cabbage cultivars [47] and 100 Indian banana germplasm [48]. When the variation of 38 Ornamental Peach germplasms was statistically analyzed, the coefficient of variation less than 10% was used as the measure of a small variation degree, 10%–20%, as a medium variation degree, and more than 20% as a high variation degree [49]. In the present study, the CV of amino acids and polyphenols ranged from 29.352% to 229.445% (Table 1). According to the above-mentioned standards, it can be seen that both amino acids and polyphenols are highly variable, which implies that the amino acids and polyphenols of the 69 cabbage varieties have a high breeding value. The Shannon–Weaver diversity index (H′) has been widely used in the evaluation of plant phenotypic traits and metabolite diversity, and it is one of the most commonly used analysis methods in the statistics of germplasm resource diversity [50,51,52]. The variation range of the 10 polyphenols in the 69 cabbage varieties was found to be higher than the 19 amino acids, because the *H*′ diversity index of amino acids varied from 1.150 to 1.341, while polyphenols were 1.184 to 1.992. The CV changes of amino acids and polyphenols also confirmed this point, which were 29.352–85.326% and 49.294–229.445%, respectively (Table 1). From the above analysis, it can be seen that the free amino acids and polyphenols of cabbages have a high level of diversity, a high degree of variation, and rich types, and both have a high utilization space and the potential for genetic improvement.

#### 2.1.3. Correlation Analysis

**Figure 1:**
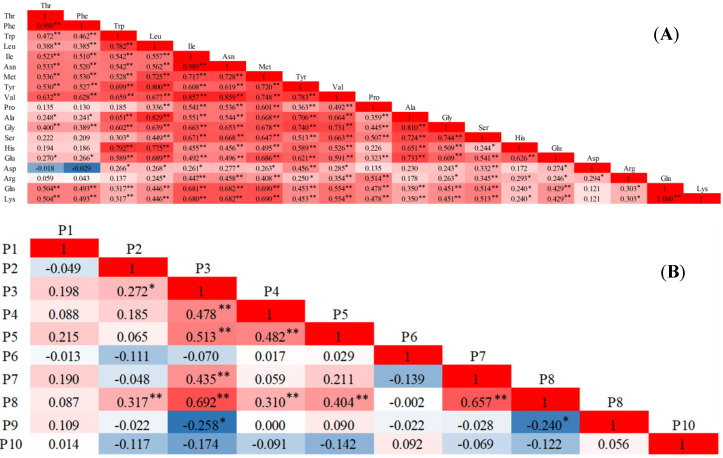
Correlation matrix based on Pearson’s correlation coefficient between different amino acids (**A**) or polyphenols (**B**). The intensity and number of colors are directly proportional to the correlation coefficient. Positive correlations are shown in red, and negative correlations are shown in blue. *, ** indicate the significance of *p* < 0.05 and *p* < 0.01, respectively. P1–P10 represent chlorogenic acid, gallic acid, 4-coumaric acid, ferulic acid, gentisic acid, cymarin, erucic acid, benzoic acid, rutin, and kaempferol, respectively.

Trp, Tyr, and Phe are precursors for the synthesis of protein and the hormone auxin [53]. Furthermore, as aromatic amino acids, they play an important role in the regulation of plant development and defense responses [54]. Glu, Gln, Asp, and Asn participate in the process of nitrogen assimilation and transport in plants. Moreover, they are also used to establish reserves during the nitrogen supply period for subsequent use in growth, defense, and reproductive processes [55]. Asp is synthesized by the transamination of oxaloacetic acid and is also used for the synthesis of Asn, Lys, Met, Thr, and Ile, and the conversion of Thr to Gly [56]. Substantial studies have shown that there is a certain internal relationship between the amino acids. A Pearson correlation analysis can reveal the degree of correlation between two parameters. After the correlation analysis of the data we obtained, we also found that there are many groups with significant or extremely significant (*p* < 0.05 or *p* < 0.01) correlation between the cabbage amino acids (Figure 1A). The correlation between Thr and Phe (*r* = 0.999, *p* < 0.01), Ile and Asn (*r* = 0.989, *p* < 0.01), and Gln and Lys (*r* = 1, *p* < 0.01) was higher. Similarly, there were multiple sets of significant or extremely significant correlations among the 10 polyphenols (Figure 1B). P2 was significantly and extremely significantly positively correlated with P3 and P8, respectively. P3 had an extremely significant positive correlation with P4 (*r* = 0.478), P5 (*r* = 0.513), P7 (*r* = 0.435), P8 (*r* = 0.692), and a significant negative correlation with P9 (*r* = −0.258). P4 had an extremely significant positive correlation with P5 and P8. P5 and P8 also showed an extremely significant positive correlation. P7 and P8 (*r* = 0.657) also showed an extremely significant positive correlation. P8 and P9 (*r* = −0.240) showed a significant negative correlation. These results indicated that there is an inherent relationship between these traits, which causes information overlap, and further reveals that principal component analysis can be performed on the basis of the analysis of the internal relationship between indicators.

#### 2.1.4. Heat Map Visualization and Hierarchical Cluster Analysis

**Figure 2:**
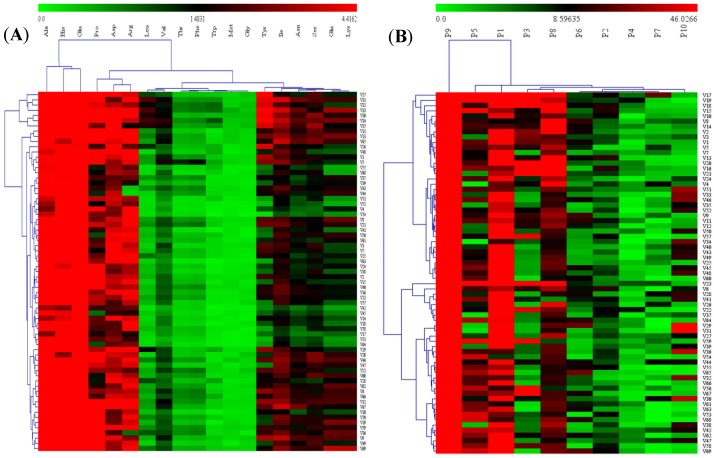
Hierarchical clustering and heat map visualization showing the content of free amino acids (**A**) and polyphenols (**B**) identified in cabbage samples. Green indicates low content. Dark indicates intermediate content, and red indicates high content. P1–P10 are defined in Figure 1.

Metabolomics has become an important technology in the fields of food quality evaluation, food processing, and food composition analysis [57,58]. Multivariate statistical technology is an important tool for metabolite analysis, including multiple linear regression analysis, discriminant analysis, hierarchical cluster analysis, principal component analysis, and factor analysis [59]. The diversity and content of amino acids and polyphenols in 69 green cabbage varieties were visualized by using hierarchical clustering and heatmap (Figure 2). As shown in Figure 2A, it can be clearly seen from the amino acid heat map visualization that among the 69 green cabbage varieties, the higher amino acids are Glu, Ala, and His; and the less abundant amino acids are Trp, Gly, and Met. According to the similarity of 19 kinds of amino acids, the 69 green cabbage varieties can be divided into two categories. The first category has seven varieties, including V27, V30, V31, V32, V33, V34, and V35. The second category has 62 varieties, including V1, V2, V3, V4, V5, V6, V7, V8, V9, V10, V11, V12, V13, V14, V15, V16, V17, V18, V19, V20, V21, V22, V23, V24, V25, V26, V28, V29, V36, V37, V38, V39, V40, V41, V42, V43, V44, V45, V46, V47, V48, V49, V50, V51, V52, V53, V54, V55, V56, V57, V58, V59, V60, V61, V62, V63, V64, V65, V66, V67, V68, and V69.

Cluster analysis and principal component analysis were used to classify polyphenols in kiwi fruit and grapefruit [60]. In the study of phenols in mango, the polyphenols were classified by cluster analysis and principal component analysis [61]. When studying onion varieties, cluster analysis is also used to classify polyphenols [62]. Similarly, in this study, the 10 polyphenols of the 69 cabbage varieties can be divided into two categories by using HCA. The first category includes varieties V1, V2, V3, V4, V5, V7, V8, V9, V10, V11, V12, V13, V14, V15, V16, V17, V18, V19, V20, V21, V24, V25, V33, V34, V35, V40, V43, V45, V46, V48, V49, V50, V51, V52, V57, and V60, a total of 36 varieties. The second category includes varieties V6, V22, V23, V26, V27, V28, V29, V30, V31, V32, V36, V37, V38, V39, V41, V42, V44, V47, V53, V54, V55, V56, V58, V59, V61, V62, V63, V64, V65, V66, V67, V68, and V69, a total of 33 varieties. It is obvious that P9 is the polyphenol with the highest content among the 69 green cabbage varieties.

#### 2.1.5. Principal Component Analysis

**Figure 3:**
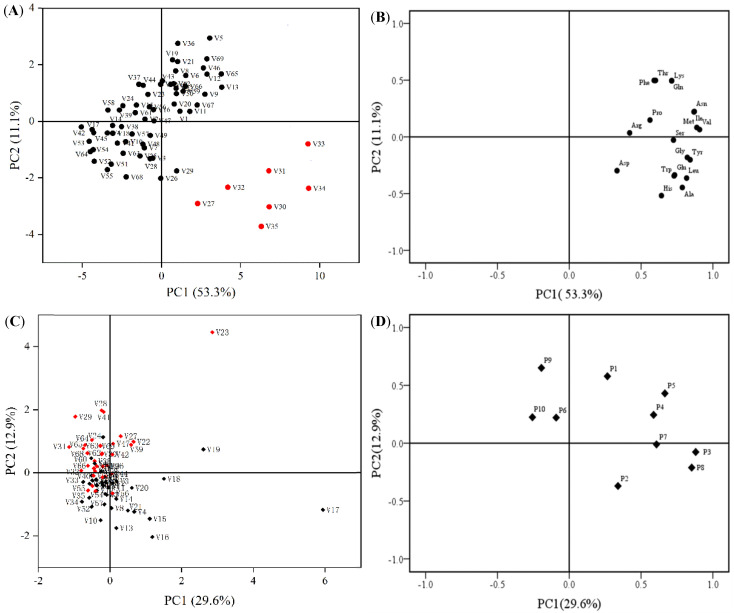
Principal component analysis (PCA) of 69 cabbage varieties and 19 free amino acids or 10 polyphenols. (**A**) shows the PCA scatter plot of amino acids. (**B**) shows a PCA loading plot of amino acids. (**C**) shows the PCA scatter plot of polyphenols. (**D**) shows a PCA loading plot of polyphenols. P1–P10 are defined in Figure 1.

Pro, which acts as a scavenger of hydroxyl radicals, is an indicator and protector of abiotic and biotic stress in plants [63,64,65]. Ala, a branched-chain amino acid, has been reported to accumulate in response to various stresses [66]. The free Phe content was observed accumulating during cadmium stress to adapt to it [67]. Many different amino acids of mature Brassica napus plants actively accumulate during the onset of drought stress [68]. The above research suggests that amino acids play a major role in plant growth and development, and adaptation to various stresses. In this experiment, 19 amino acids of 69 cabbage varieties were analyzed by PCA in order to determine the difference of information in amino acids of different varieties. From the two-dimensional principal component analysis of Figure 3A,B, it can be concluded that the 69 cabbage varieties and their 19 free amino acids formed corresponding groups. The variance contribution rate of the first two principal components can reach 64.4%, of which PC1 accounts for 53.3% of the total variance, and PC2 accounts for 11.1% of the total variance. The 69 cabbage varieties were divided into two clusters (Figure 3A), which was consistent with the results of hierarchical cluster analysis (Figure 2A). Cabbage can be divided into two categories by the first and second principal components, and all seven varieties of one category are located in the fourth quadrant. In addition, it can be concluded from Figure 3B that Val, Ile, and Asn mainly play a role in the first principal component, and Thr, Phe, Gln, and Lys mainly play a role in the second principal component.

Gonzales et al. [69] used principal component analysis to classify and distinguish polyphenols in red cabbage and Brussels sprout waste streams. The two-dimensional principal component analysis scatter plot results of the 69 cabbage varieties and 10 polyphenols showed that the two principal components explained 42.5% of the total variance, of which PC1 accounted for 29.6% of the total variance, and PC2 accounted for 12.9% of the total variance. (Figure 3C). However, since the two principal components of the 69 cabbage varieties and 10 polyphenols only explained 42.5% of the total variance, the two-dimensional PCA of polyphenols is not divided into two categories as clearly as HCA (Figure 2B). In addition, it can be concluded from Figure 3D that P3 and P8 are the main contributors of the first principal component, and P9 and P1 are the main contributors of the second principal component. Through the above scatter diagram, we could purposely discover the main amino acids and polyphenols of 69 cabbage varieties, and then determine the difference of information in the different varieties, which will be helpful for a comprehensive evaluation of the primary and secondary metabolites of cabbage.

### 2.2. Comprehensive Evaluation of Amino Acids and Polyphenol Contents of 69 Cabbage Varieties Based on PCA

Plant polyphenols are biosynthesized through the shikimic acid/phenylpropanoid pathway, and the mevalonate pathway produces terpenoids. Both of these secondary pathways produce a wide range of monomer and polymer structures that play a series of physiological and biochemical roles in plants [26]. Phenylalanine ammonia-lyase (PAL) is a key enzyme that catalyzes the deamination of the aromatic amino acid Phe to t-cinnamic acid. The latter participates in further steps by binding to coenzyme A, or hydroxylation to p-coumaric acid reaction. Alternatively, p-coumaric acid is produced directly from Tyr, the hydroxyl derivative of Phe, via tyrosine ammonia lyase, an analog of PAL [70,71,72]. It can be seen that there is a certain internal connection between amino acids and polyphenols, PCA was used to comprehensively evaluate the 19 amino acids and 10 polyphenols of 69 cabbage varieties.

In the present study, 29 indexes were selected to evaluate the nutritional value of 69 cabbage varieties. It is difficult to get a result intuitively with a simple high-low contrast method. Therefore, combined with principal component analysis, the 69 cabbage varieties can be effectively evaluated comprehensively. The data processing steps are as follows.

Firstly, the average values of amino acids and polyphenols of each sample were input into SPSS software as variables. In the process of PCA, the covariance method is selected to standardize the primitive data, which eliminates the influence of different orders of magnitude on the results. The eigenvalue and variance contribution rate are calculated, and the number of principal components with eigenvalue greater than one is determined as seven (Table S3). At the same time, the cumulative contribution rate of the first seven components to the variance was 74.386%, which represents more than 50% of the primitive indicator information, which means that we can compress it into seven principal components for further analysis.

Secondly, the related component matrix value in Table S4 showed the load of the original index in the seven principal components. According to the load values in Table S4, the indexes closely related to the first principal component were Thr, Phe, Trp, Leu, Ile, Asn, Met, Tyr, Val, Pro, Ala, Gly, Ser, His, Glu, Arg, Gln, and Lys, which implied that this component mainly represents the information of the original 18 amino acid indicators of cabbage. Asp was closely related to the fourth principal component. The second, third, fifth, sixth, and seventh main components were closely related to 10 kinds of polyphenols. Among them, the second main component was closely related to P3, P4, P5, and P8. Closely related to the third principal component was P7. Closely related to the fifth principal component were P1 and P9. Closely related to the sixth principal component was P2. Closely related to the seventh principal component were P6 and P10.

Finally, the component score coefficient matrix was also given, and the software automatically calculated the score of each component according to the matrix and standardized data (refer to Table S5). The total score was weighted by the variance contribution of each component, and the calculation formula was as follows:

$$Q_{i}=\overset{7}{\underset{m=1}{\sum }}P_{m}Z_{im}$$

where *Q* represents the total score, *i* represents the amino acid and polyphenolic compounds of Vi, m represents the main component of m, *P_m_* is the contribution rate of the main component of m to variance (see Table S3), and *Z_im_* is the main component of V_i_ amino acid and polyphenolic compound m Score. Amino acids and polyphenols were ranked in Table S5 according to the final score from highest to lowest.

In general, in Table S5, we can find that the top 10 varieties with the highest score are V33, V31, V34, V30, V35, V32, V29, V23, V65, and V19, which indicates that the contents of amino acids and polyphenols of these 10 varieties are higher. Among them, the first six varieties were consistent with the varieties screened out based on the 19 amino acids, because the comprehensive evaluation of PC1 mainly represented the information of 18 amino acids in the original 29 indicators.

## 3. Materials and Methods

### 3.1. Plant Materials

The 69 cultivars of fresh cabbage collected in this experiment were harvested on 29 September 2019, in Yuzhong, Gansu, China (35°85′ N,104°12′ E). The sample number, individual name, source details (such as company), and agronomic traits of these 69 varieties are described in Table S6. Except for the genotype differences, these cabbages, grown in the same plot, had consistent cultivation conditions and daily management measures. 3 cabbages of the same size, similar maturity, and disease-free pests were selected from each variety for the experiment. To prevent the interference of external impurities, 2 outer leaves of cabbage were stripped off. The whole cabbage was vertically divided into 4 equal parts from the growing point to the bottom, one fourth of which was immediately chopped and frozen in liquid nitrogen at −80 °C, and then the cabbage was dried in a freeze dryer (LyoQuest-85, Telstar Technologies, Barcelona, Spain). The cabbage, dried for 72 h, was ground with a grinder (TissueLyser II; QIAGEN, Hilden, Germany).

### 3.2. Chemicals

The 9 human essential amino acids, 6 conditional essential amino acids, and 4 permanent non-essential amino acids used in this experimental study were obtained from Merck and Sigma (Sigma-Aldrich GmbH, Sternheim, Germany). The 9 essential amino acids include lysine (Lys), tryptophan (Trp), phenylalanine (Phe), methionine (Met), threonine (Thr), isoleucine (Ile), Leucine (Leu), and Valine (Val). The 6 essential amino acids include arginine (Arg), asparagine (Asn), glycine (Gly), proline (Pro), tyrosine (Tyr), and glutamine (Gln). The 4 permanent non-essential amino acids include alanine (Ala), aspartic acid (Asp), serine (Ser), and glutamic acid (Glu).

The 8 phenolic acids and 2 flavonoid standard products were purchased from Shanghai Yuanye biological company, China. The 8 phenolic acids were chlorogenic acid (P1), gallic acid (P2), 4-coumaric acid (P3), ferulic acid (P4), gentilic acid (P5), artichodin (P6), erucic acid (P7), and benzoic acid (P8). The 2 flavonoids were rutin (P9) and kaempferol (P10). All other reagents were either of analytical grade or of the highest quality available.

### 3.3. Sample Preparation and Determination of Free Amino Acids

Cabbage sample preparation and LC–MS analysis of free amino acid components were carried out according to the method described by Nimbalkar et al. [73], with slight modifications. An amount of 0.1 g of frozen cabbage leaf powder was weighed and put into a 2 mL Eppendorf micro centrifuge tube, and 1 mL of 0.5 M hydrochloric acid aqueous solution was added for extraction. The sample was mixed for 20 min using a vortex mixer (MX-S, Scilogex, San Diego, CA, USA) at 8000 rpm, and then extracted by sonication (SB-800 DT, NingBo Scientz Biotechnology Co., Ltd., Ningbo, China) at 25 °C for 20 min. After sonication, a centrifuge (3-18KS, Sigma, Osterode am Harz, Germany) was used to centrifuge the sample at 20,000× *g* for 20 min. Finally, 250 μL of the extraction supernatant was transferred to the liquid chromatography sample bottle added with ISTD, and then diluted to 1 mL with 80% (*v*/*v*) acetonitrile aqueous solution.

The supernatant was passed through 0.2 µm water phase membrane filter and 5 µL of the sample was injected to LC-MS (Agilent 1290 Infinity-6460, Agilent Corp, SantaClara, CA, USA) for quantitative analysis. All separations were carried out on an Agilent InfinityLab Poroshell 120 HILIC-Z column (2.1 × 100 mm, 2.7 μm). A total of 200 mM of ammonium formate stock solution was prepared with water, and the pH was adjusted to 3 with formic acid. The mobile phase A (water phase) was prepared by diluting water and reserve solution as 9:1, and acetonitrile and reserve solution were diluted to form mobile phase B (the final ion concentration of both mobile phases was 20 mm). The column temperature was set at 25 °C, and the flow rate was 0.5 mL·min^−1^. The MS source conditions are as follows: ionization mode, ESI positive ion mode; dry gas temperature, 330 °C; gas flow rate, 13 L·min^−1^; atomizer, 35 psi; sheath gas temperature, 390 °C; sheath gas velocity, 12 L·min^−1^; capillary voltage, 1500 v.

### 3.4. Sample Preparation and Determination of Polyphenols

The sample preparation and HPLC analysis of the selected polyphenols were carried out according to the method optimized by our group. A total of 0.1 g of cabbage freeze-dried powder and 2 mL of methanol were added into a 5 mL centrifuge tube and placed at room temperature for 1 h for extraction. The samples were centrifuged at 4 °C and 8000 rpm for 10 minutes, and then the supernatant was filtered with 0.22 µ m organic phase filter membrane.

A total of 10 μL of the sample were aspirated, and symmetrical C18 column (250 mm × 4.6 mm, 5 μm, Waters Corp., Milford, MA, USA) was used for HPLC analysis. The flow rate was 1.1 mL·min^−1^, the mobile phase was methanol (A) and 1% (*v*/*v*) acetic acid (B), and the column temperature was maintained at 30 °C. Gradient elution was used. The selected 10 polyphenols included 8 phenolic acids and 2 flavonoids. Compounds were detected at 240 nm (P1, P9), 280 nm (P2, P3, and P4), and 322 nm (P5, P6, P7, P8, and P10). The compounds were identified according to the retention time of the standard products and quantified according to the standard curve. Data were analyzed using Empower Software (Waters Corp.).

### 3.5. Multivariate Statistical Analysis

Microsoft Excel 2013 was used for data statistics, and the average value (Mean), standard deviation (SD), standard error (SE), and coefficient of variation (CV) of the amino acids and polyphenols of different varieties were calculated. The Shannon–Weaver index (*H*′) was used to measure the genetic diversity of the population [52]. First, 19 amino acids and 10 polyphenols were used to classify each material into 10 grades according to the mean and standard deviation (SD). From the first grade *X_i_* < (Mean − 2SD) to the tenth grade *X_i_* ≥ (Mean + 2SD), the standard deviation of every 0.5SD was 1 grade. Then, the relative frequency of each group was calculated (pi = the number of materials in the i-th grade of a trait/total number of materials). Finally, the genetic diversity index was calculated with the formula: *H*′ = −ΣpiLnpi (Ln is the natural logarithms) [74]. Spss23.0 software (IBM Corp., Armonk, NY, United States) was used for one-way ANOVA and Pearson correlation analysis. The significant difference levels were set at *p* < 0.05 and *p* < 0.01.

Multivariate statistical analysis includes principal component analysis (PCA) and cluster analysis, both of which are used to visualize the similarity or difference in multivariate data. PCA is a method of transferring multiple variables through linear transformation to select fewer important variables [75]. PCA scores scatter plot and PCA loading plot were plotted by SPSS version 23.0 (IBM Corp., Armonk, NY, USA) and originPro 2018 (Originlab Corporation, Northampton, MA, USA), respectively. Cluster analysis and heatmap visualizations of the centered data were performed using MultiExperiment Viewer (MeV) software (version 4.8.1; Dana-Farber Cancer Institute, Boston, MA, USA).

## 4. Conclusions

The contents of 19 kinds of amino acids and 10 kinds of polyphenols in 69 varieties of green cabbage were determined. In order to make the results more intuitive and reliable, multivariate statistical techniques (including variance analysis, diversity analysis, correlation analysis, hierarchical clustering analysis, and principal component analysis) were used to process the data of amino acids or polyphenols. The experiment stated which varieties are the most or the least rich in total amino acids and total polyphenols. The diversity analysis of 19 amino acids showed that they had a high genetic diversity, and the most abundant one was Glu. The correlation analysis of 19 kinds of amino acids showed that there were multiple groups of significant or extremely significant positive correlations between them, and the correlation coefficients were all high. HCA and PCA of 19 kinds of amino acids showed that the 69 varieties of green cabbage could be divided into two categories, including seven varieties and 62 varieties. The diversity analysis of 10 polyphenols showed that they had a high genetic diversity, and the highest content was rutin (P9). There were significant or extremely significant correlations among polyphenols. The results of HCA of 10 polyphenols showed that the 69 green cabbage varieties could be divided into two categories, one category included 36 varieties, and the other had 33 varieties. Finally, based on a total of 29 indicators of amino acids and polyphenols, the 69 cabbage varieties were comprehensively evaluated and ranked by PCA. Because PC1 represents the information of 18 amino acids in the original 29 indicators, the first six varieties in the comprehensive evaluation are consistent with the varieties based on the 19 amino acid clusters. The present study could provide consumers with a theoretical basis for the selection of cabbage varieties based on amino acids and polyphenols, and it could also provide directions for the selection of improved cabbage varieties with regard to nutritional quality.

## Figures and Tables

**Table 1:** Estimates of descriptive statistics (including the min, max, mean, SD, SE, CV, and H′) for the leaf-free amino acids (mg/g dry matter) and polyphenol (μg/g dry matter) contents of 69 cabbage varieties.

Index	Min	Max	Mean	SD	SE	CV (%)	H′
Thr	0.155	1.291	0.428	0.204	0.025	47.544	1.183
Phe	0.169	1.453	0.469	0.226	0.027	48.224	1.186
Trp	0.061	1.011	0.273	0.192	0.023	70.408	1.236
Leu	0.178	2.427	0.613	0.523	0.063	85.326	1.334
Ile	0.968	4.026	2.310	0.754	0.091	32.639	1.151
Asn	0.749	2.870	1.602	0.508	0.061	31.692	1.150
Met	0.088	0.479	0.195	0.072	0.009	37.010	1.200
Tyr	0.168	5.453	1.936	1.253	0.151	64.699	1.202
Val	0.438	1.985	0.991	0.328	0.040	33.146	1.174
Pro	0.844	14.653	4.399	2.980	0.359	67.732	1.209
Ala	2.784	42.112	9.427	7.647	0.921	81.127	1.341
Gly	0.072	0.399	0.202	0.076	0.009	37.561	1.180
Ser	0.777	3.299	1.738	0.576	0.069	33.118	1.173
His	2.958	20.153	7.622	3.710	0.447	48.681	1.254
Glu	4.325	23.899	12.259	3.598	0.433	29.352	1.239
Asp	2.092	7.991	3.843	1.258	0.151	32.748	1.188
Arg	0.238	8.487	4.050	1.587	0.191	39.194	1.172
Gln	0.456	3.715	1.592	0.736	0.089	46.204	1.168
Lys	0.452	3.683	1.579	0.729	0.088	46.189	1.168
P1	6.771	179.169	59.079	37.478	4.512	63.437	1.204
P2	1.107	15.467	6.262	3.087	0.372	49.294	1.193
P3	0.897	226.888	21.449	37.773	4.547	176.108	1.551
P4	0.114	8.810	2.365	1.793	0.216	75.797	1.192
P5	0.325	413.267	29.821	58.664	7.062	196.718	1.992
P6	0.607	25.238	7.274	5.559	0.669	76.423	1.223
P7	0.055	23.212	3.083	3.601	0.434	116.810	1.284
P8	3.762	281.270	30.402	34.747	4.183	114.290	1.509
P9	45.487	1020.646	425.264	265.542	31.967	62.442	1.184
P10	0.406	180.214	12.831	29.439	3.544	229.445	1.814

SD = Standard deviation; SE = Standard error; CV = Coefficient of variation; H′ = Shannon–Weaver diversity index. P1–P10 represent chlorogenic acid, gallic acid, 4-coumaric acid, ferulic acid, gentisic acid, cymarin, erucic acid, benzoic acid, rutin, and kaempferol, respectively.

## Data Availability

Not applicable.

## Supplementary Materials

The following are available online. Table S1: Contents of 19 free amino acids and total free amino acids (mg/g dry matter) in 69 varieties of cabbage, Table S2: Contents of 10 polyphenols and total polyphenols (μg/g dry matter) in 69 varieties of cabbage, Table S3: Total variance explained, Table S4: Component matrix, Table S5: Comprehensive evaluation of 29 kinds of amino acids and polyphenols based on principal component analysis, Table S6: Basic information of 69 varieties of cabbage.

## Sample Availability

Not available.
