# Supplementary material for: Comprehensive Evaluation of Amino Acids and Polyphenols in 69 Varieties of Green Cabbage (Brassica oleracea L. var. capitata L.) Based on Multivariate Statistical Analysis
Source: Molecules. 2021 Sep 3;26(17):5355. doi: 10.3390/molecules26175355 (PMC8434452; doi:10.3390/molecules26175355)
Supplement: Supplementary file 1 [file molecules-26-05355-s001.zip › molecules-1357993-supplementary.pdf]

molecules

MDPI

Article

Comprehensive evaluation of amino acids and polyphenols in 69 varieties of green cabbage (*Brassica oleracea* L. var. *capitata* L.) based on multivariate statistical analysis

Ning Jin [1], Li Jin [1], Shilei Luo [1], Zhongqi Tang [1], Zeci Liu [1], Shouhui Wei [1], Fanhong Liu [1], Xiaoqiang Zhao [2], Jihua Yu [1,2,*] and Yuan Zhong [2,*]

[1] College of Horticulture, Gansu Agricultural University, Lanzhou 730070, China; Jinn0513@163.com (N.J.); jinli0124@163.com (L.J.); Luosl1021@163.com (S.L.); tangzq@gsau.edu.cn (Z.T.); liuzc@gsau.edu.cn (Z.L.); wlj920229@163.com (S.W.); Liufh3962@163.com (F.L.)

[2] Gansu Provincial Key Laboratory of Aridland Crop Science, Gansu Agricultural University, Lanzhou 730070, China; zhaoxq3324@163.com (X.Z.)

* Correspondence: yujihua@gsau.edu.cn (J.Y.); zhongy@gsau.edu.cn (Y.Z.)

Table S1. Contents of 19 free amino acids and total free amino acids (mg/g dry matter) in 69 varieties of cabbage.

Varieties	Thr	Phe	Trp	Leu	Ile	Asn	Met	Tyr	Val	Pro	Ala	Gly	Ser	His	Glu	Asp	Arg	Gln	Lys	Total
V1	0.594ij	0.644ij	0.314j	0.586ij	2.413g	1.684gh	0.215j	3.428f	1.321hi	4.325e	6.418d	0.230j	1.834gh	8.770c	11.675b	4.000ef	4.729e	1.510ghi	1.497ghi	56.188a
V2	0.346f	0.391f	0.206f	0.444f	1.909def	1.330ef	0.175f	2.185def	0.972ef	6.098c	4.709cd	0.2106f	1.506ef	6.132c	10.914b	3.579cde	3.715cde	1.459ef	1.447ef	47.732a
V3	0.222k	0.247k	0.228k	0.259k	2.231gh	1.515i	0.189k	2.550g	1.175j	2.526g	5.754d	0.207k	2.061h	6.267c	16.381b	5.120e	3.589f	1.084j	1.075j	52.679a
V4	0.173g	0.191g	0.099g	0.221g	1.298fg	0.892fg	0.124g	1.016fg	0.636fg	5.219d	2.784ef	0.105g	0.847fg	7.896c	11.739b	2.885ef	4.688de	1.377fg	1.366fg	43.555a
V5	1.291ef	1.453ef	0.499ef	0.564ef	3.084cde	2.139cdef	0.247f	3.048cde	1.81def	4.539c	4.413c	0.211f	1.409ef	9.239b	11.097b	3.14cde	4.182cd	1.229ef	1.218ef	54.812a
V6	0.715fg	0.792fg	0.294g	0.572g	2.797def	1.904defg	0.214g	3.148de	1.293efg	3.049de	7.712c	0.205g	1.393efg	8.189c	12.971b	3.29de	3.615d	2.16defg	2.141defg	56.451a
V7	0.369i	0.409i	0.216i	0.359i	1.995fg	1.366fgh	0.164i	2.286f	0.964hi	1.576fgh	5.597d	0.156i	1.769fgh	5.838d	13.511b	7.035c	4.069e	1.118ghi	1.108ghi	49.904a
V8	0.586f	0.648ef	0.282f	0.546f	2.364ef	1.687ef	0.208f	2.508def	1.041ef	2.689def	6.051cd	0.210f	1.603ef	6.365c	11.635b	3.916cdef	4.412cde	2.681def	2.657def	52.088a
V9	0.391h	0.432h	0.331h	0.445h	3.674efg	2.439fgh	0.230h	3.443fg	1.383gh	6.008de	9.074c	0.301h	2.247fgh	7.945cd	11.867b	4.234ef	2.927fgh	2.478fgh	2.457fgh	62.305a
V10	0.233jk	0.253jk	0.092k	0.368ijk	1.45gh	0.963ghi	0.158jk	1.59g	0.817hij	7.921c	6.731d	0.188jk	1.357gh	7.293d	12.805b	2.458f	3.680e	1.044gh	1.035gh	50.437a
V11	0.309d	0.334d	0.148d	0.602d	2.674cd	1.829d	0.288d	1.957d	1.113d	13.408b	10.313b	0.247d	2.615cd	6.233c	11.839b	5.716b	5.571c	1.935d	1.918d	69.049a
V12	0.623fg	0.678fg	0.285g	0.603fg	3.146ef	2.095efg	0.225g	3.146ef	1.515fg	6.274cd	8.656c	0.221g	2.322efg	7.376c	11.592b	4.72de	6.934cd	2.387efg	2.366efg	65.166a
V13	0.478i	0.527i	0.201i	0.752hi	3.187f	2.269fgh	0.305i	2.825fg	1.369ghi	9.130d	11.903c	0.229i	2.514fg	7.310e	14.92b	6.956e	5.866e	2.909fg	2.884fg	76.534a
V14	0.280c	0.302c	0.169c	0.240c	1.609bc	1.225bc	0.127c	1.648bc	0.805bc	2.515bc	3.311bc	0.094c	1.415bc	4.067bc	8.181b	4.425bc	3.989bc	1.023bc	1.014bc	36.439a
V15	0.408e	0.436e	0.138e	0.276e	2.252de	1.495e	0.229e	0.852e	0.932e	4.577c	4.459c	0.157e	1.206e	5.808c	10.19b	3.759cd	4.416c	1.17e	1.160e	43.922a
V16	0.427jk	0.460jk	0.152k	0.358k	2.599g	1.840h	0.157k	1.931h	0.938ij	3.113f	8.195c	0.226k	1.743h	5.537d	9.640b	4.502e	5.558d	1.386hi	1.375hi	50.136a
V17	0.330h	0.361h	0.150h	0.265h	1.14fgh	0.749gh	0.088h	0.845fgh	0.636h	1.778efg	2.962d	0.118h	0.777gh	5.931c	9.032b	2.594de	1.815ef	0.885fgh	0.878fgh	31.335a
V18	0.239ij	0.253ij	0.077j	0.296ij	1.697fg	1.199gh	0.121j	0.998ghi	0.822hij	2.507e	5.275d	0.195j	1.346gh	5.963c	8.924b	2.306ef	4.774d	0.823hij	0.816hij	38.632a
V19	0.353g	0.389g	0.113g	0.438g	2.54ef	1.877fg	0.238g	1.099fg	1.057fg	8.648c	8.127c	0.192g	1.825fg	4.819d	13.013b	2.729ef	3.883de	2.958ef	2.933ef	57.23a
V20	0.319c	0.345c	0.168c	0.231c	2.787c	1.971c	0.174c	1.207c	1.084c	4.672c	12.731b	0.331c	2.962c	2.983c	13.317b	3.992c	3.658c	2.037c	2.020c	56.988a
V21	0.778gh	0.860gh	0.296h	0.652gh	2.723defgh	1.895fgh	0.166h	2.737defgh	1.089fgh	3.906cdef	4.872cde	0.187h	1.541fgh	5.236cd	11.974b	3.463cdefg	5.640c	1.99efgh	1.973efgh	51.977a
V22	0.756hij	0.829hij	0.255ij	0.484hij	2.745ef	1.867fg	0.197j	2.485ef	1.18ghi	3.138ef	12.324b	0.334hij	1.846fg	4.809d	10.073c	3.079e	2.395ef	1.286gh	1.276gh	51.359a
V23	0.512jk	0.567jk	0.164k	0.522jk	2.217gh	1.544hij	0.172k	1.753hi	1.033ijk	3.599ef	6.691c	0.195k	1.642hij	4.803d	9.355b	3.169fg	4.269de	1.425hij	1.413hij	45.043a
V24	0.304f	0.346f	0.109f	0.394f	1.724def	1.21def	0.154f	1.119ef	0.770ef	5.157c	4.727c	0.185f	1.318def	3.972cd	8.806b	2.874cdef	3.396cde	1.453def	1.441def	39.456a
V25	0.358gh	0.384gh	0.221h	0.828gh	1.508fgh	1.088fgh	0.171h	1.896fg	0.739gh	2.571f	5.257de	0.128h	1.317fgh	9.002c	14.171b	6.151d	4.438e	1.381fgh	1.369fgh	52.98a
V26	0.380d	0.411d	0.445d	1.261d	2.101d	1.450d	0.161d	4.12cd	0.933d	2.127d	11.425b	0.210d	1.497d	9.696b	4.325cd	7.991bc	2.577d	1.147d	1.138d	53.395a
V27	0.303h	0.332h	0.298h	1.072h	1.763gh	1.262h	0.229h	5.453ef	0.915h	8.488d	23.269b	0.343h	2.408gh	7.951de	18.686c	5.571ef	4.038fg	1.311h	1.300h	84.99a
V28	0.363d	0.403d	0.530d	1.081d	1.771d	1.257d	0.147d	1.667d	0.931d	1.374d	8.417bc	0.188d	1.128d	8.920bc	11.901b	4.587cd	4.126cd	1.477d	1.464d	51.732a
V29	0.336h	0.363h	0.495h	1.462gh	2.434gh	1.689gh	0.207h	3.123fg	1.124gh	3.527efg	13.874b	0.199h	1.512gh	7.174d	11.664c	5.700de	4.905def	1.26gh	1.249gh	62.294a
V30	0.397d	0.440d	0.389d	1.831d	3.015d	2.168d	0.399d	4.272d	1.458d	7.923d	33.688b	0.399d	3.101d	18.055c	20.29c	4.444d	8.487d	1.902d	1.886d	114.544a
V31	0.567g	0.615g	0.621g	2.427fg	4.026def	2.734efg	0.273g	4.152def	1.652fg	5.523de	26.975b	0.282g	2.372fg	19.631c	18.489c	2.567fg	5.779d	2.269fg	2.249fg	103.202a
V32	0.753gh	0.817gh	0.906gh	1.441fgh	2.41defg	1.66fgh	0.265h	3.975d	1.36fgh	4.083d	24.529b	0.381h	2.012efgh	14.068c	15.439c	3.578de	3.198def	1.118gh	1.108ghi	83.099a
V33	0.728kl	0.801kl	0.87kl	2.379hij	3.943g	2.644hi	0.479l	5.131f	1.442jkl	8.609d	20.109c	0.339l	1.65ijk	20.153c	23.704b	4.369fg	6.898e	3.408gh	3.378gh	111.033a
V34	0.650kl	0.714kl	1.011jkl	2.218hij	3.698fgh	2.575ghi	0.328l	5.066f	1.985ijk	10.196e	33.034b	0.349l	2.633ghi	19.382d	23.899c	4.818f	3.836fg	2.837ghi	2.812ghi	122.041a
V35	0.507ij	0.557ij	0.623ij	1.929ghi	3.216fg	2.186gh	0.288j	4.004ef	1.704hij	2.720fgh	42.112b	0.388ij	3.299fg	13.289d	22.432c	5.242e	1.455hij	1.557hij	1.544hij	109.053a
V36	0.663fgh	0.743fgh	0.22gh	1.022efgh	2.737d	1.919def	0.243efgh	2.074def	1.092efgh	7.494c	6.482c	0.133h	1.63defg	6.768c	9.110b	2.248de	0.842efgh	2.688d	2.665d	50.773a
V37	0.613hi	0.705hi	0.153i	0.613hi	1.463efghi	1.02fghi	0.175i	1.599efghi	0.845ghi	2.295efg	8.302c	0.154i	1.391efghi	4.340d	11.866b	2.473ef	2.677e	1.837efgh	1.821efgh	44.341a
V38	0.231h	0.264gh	0.115h	0.381gh	1.805fgh	1.269fgh	0.174h	0.869gh	0.749gh	5.699cd	7.435bc	0.170h	1.87fgh	4.600de	8.775b	2.589efg	3.432ef	0.941gh	0.933gh	42.3a
V39	0.381ef	0.422ef	0.194f	0.309f	1.651ef	1.191ef	0.172f	0.892ef	0.588ef	1.605ef	6.144c	0.138f	1.419ef	4.518cd	11.530b	2.645de	1.837ef	1.442ef	1.429ef	38.505a
V40	0.628gh	0.692gh	0.217gh	0.401gh	2.566ef	1.812fg	0.216gh	1.803fg	0.991gh	5.178c	5.886c	0.176h	1.525fgh	4.769cd	13.554b	3.372de	3.532de	1.416fgh	1.404fgh	50.138a
V41	0.190e	0.212e	0.081e	0.356e	1.931de	1.339e	0.183e	0.884e	0.808e	2.008de	4.721c	0.096e	1.560e	4.925c	13.057b	4.006cd	4.100cd	0.792e	0.786e	42.034a
V42	0.275fg	0.313fg	0.066g	0.178g	1.062defg	0.76efg	0.106g	0.676efg	0.49efg	2.143cdefg	3.333c	0.107g	1.016defg	2.958cd	7.654b	2.332cdef	2.585cde	0.534efg	0.53efg	27.12a
V43	0.669e	0.741e	0.209e	0.635e	2.530e	1.730e	0.235e	1.797e	1.016e	2.035e	9.36c	0.174e	1.5210e	5.26de	13.756b	2.878de	0.303e	1.813e	1.798e	48.461a

V44	0.576i	0.638i	0.226i	0.259i	1.867ef	1.328fg	0.200i	1.713efg	0.746hi	1.241gh	5.437c	0.168i	1.377fg	5.087c	13.432b	3.752d	0.238i	2.102e	2.084e	42.471a
V45	0.216gh	0.240gh	0.105h	0.308gh	1.324efg	0.98gh	0.150h	0.897gh	0.625gh	0.844gh	5.741c	0.122h	1.154efgh	4.214d	7.567b	2.092e	1.869ef	0.774gh	0.768gh	29.99a
V46	0.565h	0.626h	0.163h	0.726h	3.071ef	2.108fg	0.322h	2.575ef	1.193gh	4.865d	13.37b	0.224h	2.876ef	4.908d	11.835c	3.436e	5.553d	2.47ef	2.449ef	63.333a
V47	0.263d	0.281d	0.252d	0.302d	2.916cd	1.538cd	0.141d	0.701d	0.883d	3.969cd	12.011b	0.228d	2.088cd	7.111bc	12.032b	3.492cd	4.075cd	1.884cd	1.868cd	56.036a
V48	0.282h	0.301h	0.474gh	0.217h	2.768e	1.914f	0.131h	1.293fg	0.857gh	5.916c	3.997d	0.187h	1.002gh	12.495b	6.124c	4.42d	5.726c	0.925gh	0.917gh	49.945a
V49	0.266h	0.286h	0.276h	0.265h	2.36fg	1.608fgh	0.127h	1.07gh	0.925gh	6.279d	8.818c	0.237h	2.396fg	8.551c	11.164b	3.194ef	4.187e	1.315gh	1.304gh	54.628a
V50	0.476gh	0.521gh	0.309h	0.712fgh	2.841f	1.919fgh	0.205h	1.163fgh	0.976fgh	10.266c	8.804cd	0.186h	1.886fgh	8.254d	12.265b	2.627fg	5.799e	1.809fgh	1.793fgh	62.811a
V51	0.181f	0.196f	0.334f	0.201f	1.465e	1.029ef	0.140f	0.608ef	0.674ef	3.61d	3.874d	0.148f	1.411e	7.042c	12.062b	3.934d	3.354d	0.654ef	0.649ef	41.565a
V52	0.191g	0.210g	0.129g	0.194g	1.535efg	1.052fg	0.105g	0.375g	0.512g	1.484efg	3.259de	0.126g	0.957fg	7.203c	12.424b	3.679d	2.7def	0.456g	0.452g	37.042a
V53	0.253j	0.275j	0.131j	0.208j	1.385g	0.984ghi	0.090j	0.419ij	0.438hij	2.529ef	5.034d	0.114j	1.09gh	6.379c	7.795b	3.001e	2.083f	0.601hij	0.596hij	33.405a
V54	0.213e	0.233e	0.061e	0.187e	0.968de	0.791de	0.127e	0.168e	0.471de	2.884cde	3.759cd	0.072e	0.888de	9.04b	11.335b	2.804cde	4.952c	0.618de	0.613de	40.187a
V55	0.253gh	0.271gh	0.392fgh	0.2gh	1.483f	1.099fgh	0.124h	0.797fgh	0.587fgh	1.320fg	4.274d	0.131h	1.217fgh	9.93c	11.219b	3.205e	2.524e	0.481fgh	0.477fgh	39.985a
V56	0.358f	0.382f	0.324f	0.418f	2.361def	1.715ef	0.169f	0.782f	0.912f	1.038f	4.827cd	0.108f	1.793ef	6.782c	12.874b	4.39cde	5.182c	2.016ef	1.999ef	48.429a
V57	0.271e	0.307e	0.158e	0.41e	2.254de	1.538de	0.129e	0.722e	0.921e	3.54d	9.037b	0.261e	1.457de	6.876c	10.050b	2.259de	3.682d	0.957e	0.949e	45.778a
V58	0.378f	0.405f	0.175f	0.391f	1.201ef	0.844ef	0.136f	0.54f	0.643f	2.252cdef	4.812cd	0.103f	1.374def	5.038c	8.304b	2.461cdef	4.371cde	1.291def	1.28def	36.000a
V59	0.645f	0.689f	0.227f	0.669f	1.964fg	1.390f	0.296f	2.141fg	0.986f	7.688c	7.443cd	0.266f	2.024fg	4.694ef	13.802b	4.919de	5.792cde	1.914fg	1.898fg	59.448a
V60	0.578jk	0.612jk	0.201k	0.457jk	2.377gh	1.66ghi	0.230k	1.449hij	0.818ijk	1.385hij	12.79b	0.234k	2.665fg	4.92d	11.018c	4.121de	3.373ef	2.261gh	2.242gh	53.391a
V61	0.417e	0.451e	0.223e	0.354e	1.971e	1.354e	0.140e	1.277e	0.674e	2.274e	4.362de	0.156d	1.168e	6.716e	12.908c	4.240b	4.301d	1.711e	1.697e	46.395a
V62	0.562i	0.594i	0.232i	0.510i	2.374fgh	1.681h	0.186i	1.759h	0.863i	1.868gh	10.125c	0.253i	1.813h	8.606d	12.605b	2.816f	5.107e	2.58fg	2.558fg	57.093a
V63	0.155f	0.169f	0.068f	0.282f	1.968def	1.345ef	0.138f	0.507f	0.650f	3.131def	5.653cd	0.128f	1.65ef	7.752c	13.721b	5.606cd	4.946cde	1.156ef	1.146ef	50.169a
V64	0.184e	0.199e	0.118e	0.182e	1.478de	0.998de	0.113e	0.542de	0.556de	1.022de	5.325c	0.110e	1.171de	7.955b	8.727b	2.574de	2.893d	0.659de	0.653de	35.459a
V65	0.451e	0.471e	0.224e	0.548e	3.890e	2.599e	0.295e	1.908e	1.449e	14.653bc	17.646b	0.246e	2.932e	3.615e	12.845c	4.103e	7.570d	2.141e	2.122e	79.711a
V66	0.709fgh	0.755fgh	0.497gh	0.446gh	2.711de	1.909efg	0.183h	1.815efgh	1.03efgh	2.210ef	8.082c	0.223gh	1.969efg	8.047c	14.51b	4.031d	3.845d	2.260ef	2.240ef	57.471a
V67	0.443d	0.470d	0.301d	0.546d	3.915cd	2.870cd	0.204d	2.486cd	1.333d	5.615cd	5.957cd	0.209d	1.868d	8.241bc	12.998b	5.814cd	6.622cd	1.668d	1.654d	63.213a
V68	0.193f	0.202f	0.283f	0.232f	1.993def	1.426def	0.125f	0.916def	0.984def	1.569def	7.420c	0.181f	1.715def	11.651b	11.405b	3.480d	3.166de	0.501ef	0.497ef	47.941a
V69	0.459j	0.484j	0.311j	0.791ij	3.043g	2.086h	0.257j	1.707h	1.083i	8.126d	10.437c	0.244j	2.053h	10.557bc	11.067b	3.186fg	4.737e	3.715f	3.683f	68.026a

Mean values (n = 3) indicated by the same letters in a line do not significantly different at 5% level using Duncan's multiple range test.

Table S2. Contents of 10 polyphenols and total polyphenols (μg/g dry matter) in 69 varieties of cabbage.

Varieties	P1	P2	P3	P4	P5	P6	P7	P8	P9	P10	Total
V1	85.889c	6.238ef	23.418de	0.691f	22.805de	11.224def	2.823f	25.234d	166.934b	2.588f	347.843a
V2	83.993bc	4.249d	28.172cd	1.71d	14.041d	11.145d	2.201d	47.865bcd	98.829b	2.801d	295.007a
V3	96.376c	6.384e	26.140d	3.13e	3.929e	6.162e	3.863e	20.804d	115.259b	5.641e	287.688a
V4	23.317c	6.992c	10.189c	3.575c	0.567c	3.265c	8.623c	78.608c	264.436b	4.323c	403.895a
V5	27.757c	1.107c	14.683c	1.115c	18.989c	7.362c	7.263c	34.865c	162.068b	2.238c	277.447a
V6	33.519b	15.467b	15.819b	2.06b	18.637b	8.140b	0.061b	32.154b	1020.646a	7.906b	1154.41a
V7	50.126c	4.626c	2.183c	2.870c	9.718c	2.495c	4.637c	24.651c	149.287b	1.72c	252.311a
V8	9.828d	5.314d	11.206d	1.960d	15.937d	16.186d	5.068d	55.634c	100.099b	5.806d	227.038a
V9	60.150c	3.910c	17.189c	1.252c	13.567c	18.39c	5.215c	31.294c	200.521b	1.626c	353.114a
V10	44.502b	8.703cd	13.438cd	0.273d	7.821cd	6.401cd	2.896cd	24.241c	51.073b	2.492cd	161.841a
V11	38.534c	8.131c	13.836c	4.132c	22.095c	14.717c	0.209c	26.031c	218.214b	2.738c	348.636a
V12	69.032c	5.928c	13.962c	0.434c	4.897c	8.056c	1.965c	29.217c	239.378b	3.054c	375.922a
V13	48.849c	15.157c	14.103c	1.249c	10.988c	10.834c	0.990c	49.595c	140.951b	0.804c	293.52a
V14	19.282cd	5.036d	14.882d	4.336d	18.985cd	10.696d	2.111d	49.908c	116.574b	3.878d	245.69a
V15	30.96de	13.848ef	11.391ef	3.558f	122.162b	4.941f	2.145f	78.963c	45.487d	1.949f	315.403a
V16	26.961d	14.942de	70.229c	4.096e	28.342d	1.963e	2.801e	60.202c	122.194b	2.246e	333.977a
V17	90.903e	10.583f	226.888c	4.890f	153.575d	6.427f	23.212f	281.27b	170.052d	2.559f	970.357a
V18	146.809b	13.139e	113.151c	4.525e	16.099e	9.133e	1.305e	47.177d	150.596b	1.389e	503.322a
V19	47.975d	6.262e	149.746c	8.810e	240.398b	7.032e	1.721e	36.578d	58.932d	0.406e	557.860a
V20	62.248c	4.46de	63.560c	4.458de	19.371de	5.558de	1.275e	46.094cd	117.605b	0.991e	325.620a
V21	35.574cd	6.894d	63.288c	3.378d	3.364d	6.086d	2.007d	47.231cd	125.086b	2.067d	294.975a
V22	73.653b	8.871b	42.409b	8.087b	10.995b	4.150b	1.025b	25.757b	810.139a	0.692b	985.778a
V23	153.482d	4.565e	69.773de	5.895e	413.267c	7.499e	6.243e	74.738de	933.246b	2.354e	1671.063a
V24	179.169c	2.561e	7.109de	0.360e	21.248de	4.991e	1.864e	33.595d	271.585b	6.662de	529.142a
V25	57.406bc	3.936c	3.081c	2.127c	18.461c	2.742c	3.940c	23.563c	280.701ab	1.659c	397.616a
V26	49.975c	7.135d	2.060d	0.847d	7.764d	5.360d	7.859d	43.691c	883.596b	2.134d	1010.422a
V27	146.184c	6.811d	4.195d	3.985d	6.400d	3.169d	4.392d	35.541d	573.396b	8.332d	792.404a
V28	136.984c	2.911d	2.522d	0.958d	7.305d	11.847d	9.511d	14.096d	825.881b	15.979d	1027.995a
V29	67.810d	4.671e	2.623e	3.47e	1.671e	24.489e	0.122e	22.098e	458.659b	165.703c	751.316a
V30	54.087c	4.442c	8.034c	0.798c	8.014c	5.089c	11.095c	18.885c	763.636b	37.565c	911.644a
V31	60.652cd	5.875d	2.519d	1.305d	1.006d	2.132d	0.614d	14.477d	477.429b	180.214c	746.224a
V32	21.180c	1.64c	3.932c	0.899c	19.503c	5.705c	2.741c	15.948c	509.53b	28.845c	609.923a
V33	52.523c	3.166d	2.945d	1.259d	5.850d	3.753d	0.643d	13.639d	248.988b	27.016cd	359.781a
V34	17.834c	5.888c	5.544c	0.252c	0.325c	3.088c	2.371c	11.231c	357.722b	24.921c	429.177a
V35	38.394c	4.344d	4.388d	0.763d	2.723d	1.754d	3.208d	15.001d	264.109b	9.003d	343.687a
V36	19.35cd	7.421d	5.277d	1.193d	19.857cd	2.263d	3.282d	21.888cd	577.566b	37.797c	695.894a
V37	62.156c	8.337c	4.427c	0.554c	0.797c	2.309c	4.197c	19.113c	829.290b	5.805c	936.985a
V38	89.875b	6.978b	4.547b	0.857b	2.002b	1.710b	1.395b	32.999b	699.222a	16.159b	855.746a
V39	57.963c	5.639c	5.233c	3.563c	4.001c	1.872c	2.930c	21.484c	582.576b	8.996c	694.256a
V40	58.658b	2.649b	2.566b	1.156b	6.962b	2.878b	4.938b	11.792b	350.621a	12.486b	454.708a
V41	154.652c	8.412d	7.663d	2.148d	26.653d	7.790d	3.205d	3.762d	866.261b	17.208d	1097.754a
V42	38.309c	4.667d	4.695d	4.331d	36.477c	2.232d	6.101d	22.805cd	708.132b	12.549d	840.300a
V43	58.413b	5.509b	3.910b	0.422b	13.160b	2.337b	2.533b	15.558b	388.781a	14.842b	505.464a
V44	48.803c	5.969c	12.23c	2.023c	11.669c	11.148c	9.811c	17.926c	438.662b	13.101c	571.342a
V45	54.408c	6.454c	3.160c	1.586c	27.028c	12.145c	1.509c	23.037c	288.920b	11.896c	430.144a
V46	62.305c	6.536d	2.554d	0.489d	24.127cd	11.469d	2.998d	17.672cd	251.692b	26.978cd	406.819a
V47	85.483c	5.943d	3.578d	4.439d	16.796cd	3.641d	6.491d	11.402cd	651.484b	13.998cd	803.256a
V48	59.144c	7.127d	3.18d	2.284d	15.244cd	4.979d	1.345d	15.409cd	272.035b	19.421cd	400.166a

V49	97.883c	3.195c	3.419c	0.397c	8.996c	2.935c	4.611c	21.224c	382.435b	16.342c	541.437a
V50	62.572c	2.797c	5.454c	4.648c	5.285c	3.331c	0.980c	17.584c	212.124b	15.15c	329.925a
V51	23.727c	4.547c	5.527c	3.617c	20.303c	6.659c	3.661c	24.845c	237.890b	31.578c	362.355a
V52	38.406c	6.887fgh	8.84fg	0.114h	10.746ef	2.429gh	1.507gh	26.883d	246.444b	16.855e	359.111a
V53	25.618c	8.689c	5.854c	1.799c	40.596c	0.607c	0.176c	14.086c	792.838b	1.843c	892.106a
V54	6.771c	7.128c	3.597c	2.373c	39.089c	0.885c	0.535c	18.864c	554.297b	1.403c	634.943a
V55	32.037c	8.570c	3.734c	0.796c	24.473c	10.654c	0.063c	13.633c	440.326b	0.690c	534.975a
V56	16.035c	6.489d	66.28d	1.291d	35.07d	3.751d	4.182d	7.194d	482.738b	1.380d	624.411a
V57	65.911c	9.033de	44.504cd	0.548e	12.085de	3.648de	0.087e	15.882de	220.102b	1.136e	372.935a
V58	52.668c	8.047d	23.473cd	1.681d	28.454cd	0.741d	0.368d	24.006cd	646.354b	1.004d	786.796a
V59	115.189c	4.278e	85.581cd	2.505e	45.027de	1.689e	5.011e	5.981e	507.072b	2.180e	774.512a
V60	91.684c	4.205e	1.112e	2.086e	21.986d	9.925e	0.198e	8.220e	317.159b	1.306e	457.880a
V61	12.274d	6.721def	1.193fg	4.051efg	25.332c	7.808de	0.073g	23.230c	760.777b	1.225fg	842.683a
V62	47.44c	6.779c	9.732c	4.145c	28.202c	10.903c	0.203c	19.421c	687.449b	2.690c	816.962a
V63	41.579c	3.000c	0.897c	2.140c	25.696c	11.740c	0.102c	20.848c	786.275bc	0.706c	892.982a
V64	44.872b	5.73b	4.125b	3.261b	34.865b	14.625b	0.185b	11.529b	822.217a	1.784b	943.192a
V65	44.675c	2.174c	1.693c	1.623c	32.580c	25.238c	1.05c	13.014c	437.563b	0.559c	560.169a
V66	46.027c	3.468c	1.905c	2.234c	20.554c	5.891c	0.204c	14.942c	520.646b	2.057c	617.928a
V67	31.079cd	1.927d	55.189c	0.83d	22.992cd	12.212d	0.374d	18.896cd	475.022b	2.085d	620.608a
V68	17.680c	4.119c	4.944c	1.794c	39.912c	15.480c	0.055c	27.419c	789.442b	0.410c	901.256a
V69	70.879c	8.496c	1.294c	2.721c	39.823c	21.996c	0.336c	25.329c	625.956b	1.388c	798.219a

Mean values (n = 3) indicated by the same letters in a line do not significantly different at 5% level using Duncan's multiple range test.

Table S3. Total variance explained.

Component	Initial Eigenvalues			Extraction Sums of Squared Loadings		
	Total	% of Variance	Cumulative %	Total	% of Variance	Cumulative %
1	10.494	36.185	36.185	10.494	36.185	36.185
2	3.022	10.419	46.604	3.022	10.419	46.604
3	2.310	7.964	54.568	2.310	7.964	54.568
4	1.991	6.866	61.434	1.991	6.866	61.434
5	1.355	4.672	66.107	1.355	4.672	66.107
6	1.236	4.260	70.367	1.236	4.260	70.367
7	1.165	4.019	74.386	1.165	4.019	74.386
8	0.971	3.349	77.734			
9	0.902	3.109	80.843			
10	0.851	2.935	83.778			
11	0.819	2.825	86.603			
12	0.677	2.333	88.936			
13	0.535	1.845	90.782			
14	0.514	1.772	92.553			
15	0.475	1.637	94.190			
16	0.389	1.342	95.532			
17	0.263	0.906	96.438			
18	0.237	0.817	97.255			
19	0.172	0.594	97.849			
20	0.152	0.526	98.375			
21	0.135	0.466	98.841			
22	0.114	0.394	99.235			
23	0.083	0.285	99.520			
24	0.069	0.240	99.759			

25	0.046	0.159	99.918
26	0.016	0.055	99.973
27	0.007	0.025	99.998
28	0.000	0.002	100
29	0.000	0.000	100

Extraction Method: Principal Component Analysis.

Table S4. Component matrix.

Index	Component						
	1	2	3	4	5	6	7
Thr	0.587	0.238	0.130	−0.706	0.035	−0.110	0.062
Phe	0.576	0.235	0.142	−0.714	0.034	−0.116	0.058
Trp	0.745	−0.261	0.373	−0.091	−0.065	0.099	0.169
Leu	0.826	−0.188	0.370	0.114	0.081	0.188	0.020
Ile	0.866	0.172	−0.236	−0.038	−0.015	0.070	0.092
Asn	0.869	0.175	−0.243	−0.047	−0.031	0.077	0.098
Met	0.882	0.119	−0.058	0.009	0.035	−0.023	−0.095
Tyr	0.838	−0.022	0.274	0.039	−0.078	−0.118	0.065
Val	0.901	0.146	0.042	−0.060	−0.077	−0.059	0.090
Pro	0.551	0.256	−0.346	0.303	−0.056	0.027	−0.100
Ala	0.789	−0.177	0.281	0.280	0.148	−0.008	−0.211
Gly	0.809	0.070	0.126	0.193	0.122	−0.195	−0.174
Ser	0.723	0.084	−0.227	0.301	0.124	−0.283	−0.190
His	0.657	−0.342	0.356	0.139	−0.101	0.330	0.021
Glu	0.737	−0.138	0.205	0.179	−0.033	−0.026	−0.265
Asp	0.336	−0.108	−0.068	0.354	−0.326	−0.322	0.219
Arg	0.418	0.131	−0.358	0.368	−0.121	0.150	0.139
Gln	0.694	0.381	−0.295	−0.188	0.082	0.101	−0.072
Lys	0.694	0.381	−0.295	−0.188	0.082	0.101	−0.072
P1	−0.093	0.195	0.128	0.342	0.507	−0.414	0.048
P2	−0.243	0.273	−0.003	0.036	−0.200	0.409	−0.432
P3	−0.205	0.782	0.310	0.160	−0.072	0.083	−0.035
P4	−0.262	0.546	−0.006	0.171	0.292	0.238	−0.081
P5	−0.196	0.604	0.158	0.106	0.404	0.144	0.122
P6	0.124	0.129	−0.461	0.159	0.015	0.052	0.654
P7	−0.121	0.376	0.652	0.087	−0.146	−0.276	0.216
P8	−0.283	0.650	0.473	0.148	−0.290	0.084	0.145
P9	−0.099	−0.317	0.118	−0.094	0.668	0.087	0.096
P10	0.340	−0.277	0.213	0.156	0.153	0.495	0.324

Extraction Method: Principal Component Analysis. P1-P10 are defined in Table 1. 7 components extracted.

Table S5. Comprehensive evaluation of 29 kinds of amino acids and polyphenols based on principal component analysis.

Varieties	Score of PC1	Score of PC2	Score of PC3	Score of PC4	Score of PC5	Score of PC6	Score of PC7	Total Score	Ranking
V1	0.380	0.158	−0.041	−0.359	−0.634	−0.896	0.655	0.085	26
V2	−0.296	0.185	−0.289	0.329	−0.586	−0.735	0.458	−0.129	39
V3	−0.171	−0.139	0.059	1.148	−0.659	−1.248	−0.403	−0.093	37
V4	−1.096	0.113	0.434	0.471	−1.373	0.817	−0.283	−0.359	57
V5	0.968	0.626	0.929	−3.547	−1.068	−1.385	1.883	0.213	18
V6	0.391	0.278	−0.064	−1.728	0.475	1.478	−0.773	0.101	23
V7	−0.313	−0.427	0.131	0.389	−1.365	−1.432	0.148	−0.240	48
V8	0.297	0.724	−0.541	−1.049	−1.247	0.057	1.153	0.058	29
V9	0.878	0.618	−0.871	0.146	−0.520	−0.989	1.261	0.307	14
V10	−0.651	−0.444	−0.225	0.500	−1.231	0.129	−1.127	−0.363	59
V11	0.535	0.709	−1.893	1.555	−0.688	−0.025	−0.309	0.178	21
V12	0.904	0.524	−0.989	−0.338	−0.811	−0.783	0.391	0.224	16
V13	1.103	1.096	−1.482	0.689	−1.803	0.211	−1.016	0.327	13
V14	−0.936	−0.015	−0.481	0.163	−1.189	0.132	0.837	−0.384	61
V15	−0.607	1.008	−0.147	−0.015	−1.208	1.323	−0.835	−0.161	44
V16	−0.294	1.120	−0.072	0.259	−1.570	0.987	−1.405	−0.065	36
V17	−1.806	4.102	4.381	1.061	−1.817	0.361	1.343	0.180	20
V18	−1.080	1.029	0.131	1.168	0.124	0.529	−0.900	−0.201	46
V19	−0.021	3.227	−0.693	0.630	1.281	1.639	−1.022	0.405	10
V20	0.193	1.036	−0.775	0.874	0.079	−1.041	−1.212	0.087	24
V21	0.264	1.073	−0.161	−1.417	−1.015	0.207	0.060	0.061	28
V22	0.144	0.855	0.407	−1.125	1.823	−0.100	−1.082	0.134	22
V23	−0.540	2.513	1.139	0.495	4.015	−0.084	1.430	0.433	8
V24	−0.707	−0.173	−0.265	0.317	1.020	−1.713	0.178	−0.292	53
V25	−0.383	−0.700	0.363	0.327	−0.907	−0.672	0.132	−0.226	47
V26	−0.027	−0.894	1.344	0.271	−0.637	−1.064	1.429	0.005	33
V27	0.636	−0.274	1.117	2.228	1.013	−1.815	−1.441	0.356	12
V28	−0.175	−0.863	1.102	0.299	1.080	−1.101	1.975	0.038	31
V29	0.531	−1.083	0.078	1.377	0.530	2.163	3.800	0.450	7
V30	2.110	−0.513	1.317	2.213	0.428	−0.199	−0.533	0.957	4
V31	2.286	−1.169	1.463	0.586	0.983	3.600	0.578	1.084	2
V32	1.374	−1.216	1.956	−0.781	0.178	−0.300	0.330	0.481	6
V33	2.896	−0.184	0.707	−0.247	−0.363	1.250	−0.442	1.087	1
V34	2.901	−0.670	1.391	0.149	−0.712	0.721	−1.166	1.052	3
V35	1.976	−1.086	1.848	0.797	−0.149	−1.329	−1.890	0.664	5
V36	0.351	0.232	−0.153	−2.130	0.406	1.031	−0.533	0.034	32
V37	−0.436	−0.387	0.431	−1.676	0.840	−0.292	−0.898	−0.288	52
V38	−0.749	−0.677	−0.221	0.389	0.777	−0.283	−0.847	−0.342	56
V39	−0.811	−0.439	0.179	−0.782	0.752	−0.036	−0.836	−0.379	60
V40	0.044	−0.191	−0.049	−1.155	−0.103	−1.062	0.097	−0.133	40
V41	−0.854	−0.672	−0.178	0.968	1.572	−0.580	0.109	−0.274	51
V42	−1.556	−0.582	0.487	−0.388	0.807	0.082	−0.056	−0.573	66
V43	0.068	−0.299	0.363	−1.947	0.293	−0.659	−0.729	−0.155	43
V44	−0.337	0.124	0.522	−1.480	0.029	−0.862	0.460	−0.186	45
V45	−1.246	−0.740	−0.199	−0.213	0.030	0.207	0.320	−0.535	65
V46	0.871	0.490	−1.151	−0.257	0.085	−0.514	0.037	0.240	15
V47	−0.173	−0.002	−0.192	0.625	1.152	−0.188	−0.563	−0.012	34
V48	−0.313	−0.652	−0.387	0.461	−0.947	0.981	0.761	−0.152	42

V49	−0.157	−0.557	−0.153	0.770	0.269	−1.089	−0.232	−0.117	38
V50	0.311	0.239	−0.875	0.073	0.227	0.451	−0.429	0.085	25
V51	−0.908	−0.830	0.158	0.630	−0.701	0.448	0.314	−0.360	58
V52	−1.225	−1.256	0.248	0.119	−1.090	0.227	−0.477	−0.607	67
V53	−1.389	−1.122	0.096	−0.462	0.407	0.767	−0.950	−0.630	69
V54	−1.356	−1.086	−0.086	0.017	−0.390	1.298	−0.883	−0.608	68
V55	−0.985	−1.335	0.112	−0.127	−0.593	0.704	0.077	−0.490	64
V56	−0.238	0.062	−0.313	−0.331	−0.651	0.415	−0.125	−0.145	41
V57	−0.554	−0.337	−0.181	0.179	−0.364	0.203	−1.257	−0.296	54
V58	−1.046	−0.469	−0.094	−0.647	0.433	0.540	−0.916	−0.473	62
V59	0.356	0.932	0.102	0.115	0.810	−1.629	−0.591	0.187	19
V60	0.218	0.073	−0.904	−0.564	0.890	−1.367	−0.254	−0.051	35
V61	−0.511	−0.384	−0.518	−0.602	0.248	0.889	−0.020	−0.259	50
V62	0.211	0.298	−0.718	−0.781	1.137	0.760	−0.274	0.071	27
V63	−0.682	−0.973	−0.957	0.997	0.198	−0.163	0.798	−0.321	55
V64	−1.281	−1.033	−0.403	0.107	0.990	0.789	0.709	−0.488	63
V65	1.233	0.733	−2.765	1.123	0.217	−0.707	1.593	0.424	9
V66	0.495	−0.161	−0.292	−1.465	0.383	−0.392	0.072	0.043	30
V67	0.744	0.165	−1.226	0.280	−0.866	−0.323	1.668	0.221	17
V68	−0.622	−1.227	−0.145	0.473	0.224	0.494	1.079	−0.257	49
V69	0.865	0.916	−1.847	−0.153	1.052	1.167	0.576	0.373	11

Total score was weighted by the contribution of each PC to variance. PC principal component.

Table S6. Basic information of 69 varieties of cabbage.

Number	Variety name	origin	Leaf color	Spherical shape	Compactness
V1	GA1827	Caiyuan Seed Shop, Yuexiu District, Guangzhou	Green	circular type	Tight
V2	Zhonggan595	Institute of Vegetables and Flowers, Chinese Academy of Agricultural Sciences	Green	circular type	Tight
V3	Houteng50	Shandong Lufa Agricultural Technology Co., Ltd.	Green	circular type	Tight
V4	Jiaoyang	Shanxi Qianyousheng Agricultural Technology Co., Ltd.	Green	circular type	Medium
V5	Zhonggan828	Institute of Vegetables and Flowers, Chinese Academy of Agricultural Sciences	Green	circular type	Tight
V6	GL1903	Tianjin Gengyun Seed Industry Co., Ltd.	Green	circular type	Loose
V7	Meicui1	Hebei Xingtai Jinshi Seed Industry Co., Ltd.	Green	Elliptic type	Tight
V8	14468	Beijing Jielia Seed Industry Co., Ltd.	Green	circular type	Medium
V9	Xingshu25	Lanzhou Runfeng Seed Industry Co., Ltd.	Green	circular type	Medium
V10	Yu-4	Shantou Xintiandi Agriculture Co., Ltd.	Green	circular type	Loose
V11	Lvbao55	Xingtai Dali Seedling Company	Green	Elliptic type	Tight
V12	17gl-3008	Sichuan Zhongdu Hi-Tech Seed Industry Co., Ltd.	Green	circular type	Tight
V13	Meidi60MS	Xingtai Chenhui Seed Industry Co., Ltd.	Green	circular type	Tight
V14	Duofulvguan1	Tianjin Jinmu Maohe Agricultural Technology Co., Ltd.	Green	circular type	Medium
V15	1836	Beijing Jingyan Yinong Seed Industry Technology Co., Ltd.	Green	circular type	Loose
V16	Chunbao5	Hebei Xingtai Tianfeng Seed Industry Co., Ltd.	Green	circular type	Medium
V17	Lixin266	Fuzhou Lixin Seedling Co., Ltd.	Green	circular type	Tight
V18	Bicui6022	Lanzhou Baofeng Seedling Co., Ltd.	Green	circular type	Tight
V19	Dingfeng	Lvheng Technology Group Co., Ltd.	Green	circular type	Tight
V20	GA1826	Caiyuan Seed Shop, Yuexiu District, Guangzhou	Green	Elliptic type	Medium
V21	19-1523	Ningxia Jufeng Seedling Co., Ltd.	Green	circular type	Medium
V22	Ganlan-6	Lanzhou Tianyuan Seedling Co., Ltd.	Green	circular type	Medium
V23	GS-19-007	Xingtai Jialihe Seedling Co., Ltd.	Green	circular type	Medium
V24	Tietou65	Beijing Huanai Agricultural Development Co., Ltd.	Green	circular type	Tight
V25	Lvying50	Xingtai Dali Seedling Company	Green	circular type	Tight
V26	Lvxiu	Henan Yuyi Seed Industry Technology Development Co., Ltd.	Green	circular type	Tight
V27	ZaohuoB	Hubei Xiangyang photosynthesis fruit and vegetable Co., Ltd	Green	circular type	Loose
V28	ZSGL-01	Gansu Zeshi Agricultural Technology Co., Ltd.	Green	circular type	Tight
V29	Letu	Shanxi Qianyousheng Agricultural Technology Co., Ltd.	Green	circular type	Medium
V30	Shuoyuan2	Beijing Shuoyuan Seed Co., Ltd.	Dark green	circular type	Medium

V31	Jingdinghaoyue	Lanzhou Dongping Seed Co., Ltd.	Green	circular type	Tight
V32	Lvsejingdian	Beijing Dele Runcheng Agricultural Technology Development Co., Ltd.	Green	circular type	Tight
V33	1802	Beijing Jingyan Yinong Seed Industry Technology Co., Ltd.	Green	circular type	Tight
V34	Lvxiu2	Henan Yuyi Seed Industry Technology Development Co., Ltd.	Green	circular type	Tight
V35	GL1901	Tianjin Gengyun Seed Industry Co., Ltd.	Green	circular type	Tight
V36	Shunyuan	Hebei Xingtai City Xingpai Vegetable Seed Co., Ltd.	Green	circular type	Loose
V37	Shenlong70	Hebei Xingtai Shenlong Seed Industry Co., Ltd.	Green	circular type	Tight
V38	Jiutouniao23	Wuhan Jiutou Bird Seedling Co., Ltd.	Light green	circular type	Tight
V39	Tian53	Xingtai Dali Seedling Company	Dark green	circular type	Medium
V40	Tian55	Xingtai Jialihe Seedling Co., Ltd.	Green	circular type	Tight
V41	Lvxianfeng	Beijing Dele Runcheng Agricultural Technology Development Co., Ltd.	Green	circular type	Tight
V42	17gl-2016	Sichuan Zhongdu Hi-Tech Seed Industry Co., Ltd.	Green	circular type	Medium
V43	58	Xingtai Dali Seedling Company	Green	circular type	Loose
V44	GL1824	Guangzhou Xingtian Seed Industry Co., Ltd.	Green	circular type	Tight
V45	Chunguan	Shanghai Huiyang Seedling Co., Ltd.	Dark green	circular type	Medium
V46	GL1904	Tianjin Gengyun Seed Industry Co., Ltd.	Dark green	circular type	Medium
V47	Xidinong	Tianjin Jinmu Maohe Agricultural Technology Co., Ltd.	Green	circular type	Tight
V48	MR-ZL(K2-24)	Handan Manrun Agricultural Technology Co., Ltd.	Green	circular type	Tight
V49	17gl-2011	Sichuan Zhongdu Hi-Tech Seed Industry Co., Ltd.	Green	circular type	Tight
V50	Gan56	Beijing Jingyan Yinong Seed Industry Technology Co., Ltd.	Green	circular type	Tight
V51	Lvqiu	Ningxia Jufeng Seedling Co., Ltd.	Green	circular type	Tight
V52	Ganlan-4	Lanzhou Tianyuan Seedling Co., Ltd.	Green	circular type	Medium
V53	Zhenbao	Shanghai Huihe Seed Industry Co., Ltd.	Green	circular type	Loose
V54	9Hao	Shanxi Qianyousheng Agricultural Technology Co., Ltd.	Green	circular type	Tight
V55	17gl-2014	Sichuan Zhongdu Hi-Tech Seed Industry Co., Ltd.	Green	circular type	Tight
V56	Jiulv55	Handan Manrun Agricultural Technology Co., Ltd.	Green	circular type	Tight
V57	Wg-1703	Shanghai Huihe Seed Industry Co., Ltd.	Green	circular type	Tight
V58	Fugui	Henan Yuyi Seed Industry Technology Development Co., Ltd.	Green	circular type	Medium
V59	24Hao	Shanxi Qianyousheng Agricultural Technology Co., Ltd.	Green	circular type	Tight
V60	Xinshuxinxiu	Ningxia Jufeng Seedling Co., Ltd.	Green	circular type	Medium
V61	MR-ZL(K2-25)	Handan Manrun Agricultural Technology Co., Ltd.	Green	circular type	Tight
V62	Fuqiang	Henan Yuyi Seed Industry Technology Development Co., Ltd.	Green	circular type	Loose
V63	Aoli58	Wuhan Hanyan Seedling Co., Ltd.	Green	circular type	Tight

V64	17gl-3006	Sichuan Zhongdu Hi-Tech Seed Industry Co., Ltd.	Green	circular type	Medium
V65	Xueyin5002	Hubei Xueyin Agricultural Technology Co., Ltd.	Green	circular type	Tight
V66	Manyue506	Beijing Huanai Agricultural Development Co., Ltd.	Green	circular type	Tight
V67	A0166	Fujian Minhui Seed Co., Ltd.	Green	circular type	Loose
V68	Jipinyihao	Xingtai Chenhui Seed Industry Co., Ltd.	Green	circular type	Tight
V69	Desaijiali	Shaanxi Desai Seed Industry Co., Ltd.	Green	circular type	Tight
